# Supplementary material for: Cryo-EM structure of renal AL amyloid fibrils from a patient with λ1 light chain amyloidosis
Source: Nat Commun. 2025 Dec 17;17:848. doi: 10.1038/s41467-025-67556-0 (PMC12827339; doi:10.1038/s41467-025-67556-0)
Supplement: Supplementary file 1 — Supplementary Information [file 41467_2025_67556_MOESM1_ESM.pdf]

Supplementary Information

Cryo-EM structure of renal AL amyloid fibrils from a patient with λ1 light chain amyloidosis

Chenyue Yu, Yeyang Ma et al.

Supplementary Figure 1

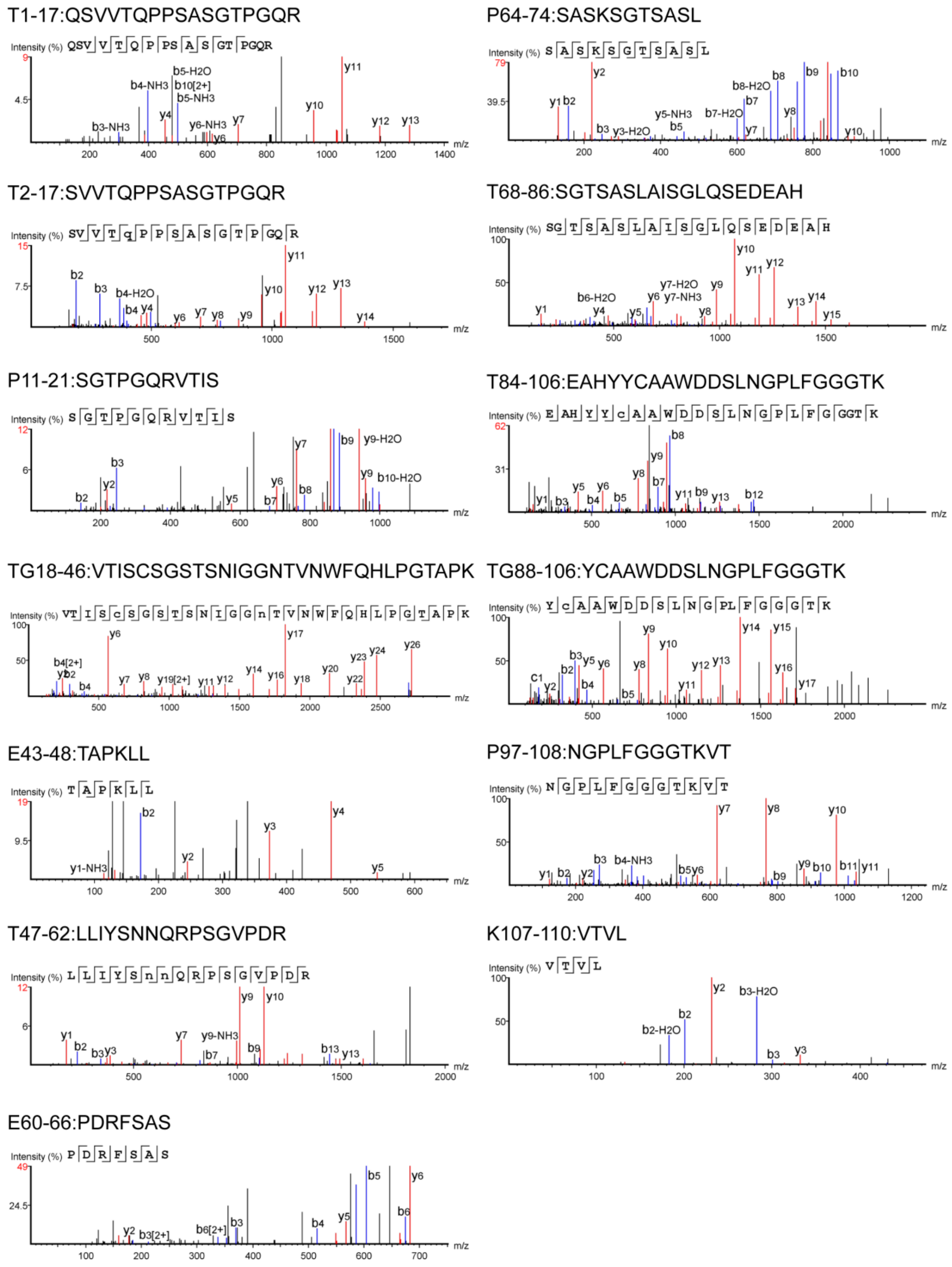

QSVVTQPPSASGTPGQRVTISCSGSTSNIGGNTVNWFQHLPGTAPKLLIYSNNQR
PSGVPDRFSASKSGTSASLAISGLQSEDEAHYYCAAWDDSLNGPLFGGGTKVTVL

Supplementary Figure 1

The secondary mass spectrum and protein sequence of IGLV1-44.

The secondary mass spectrum was generated by the PEAKS Studio software.

Supplementary Figure 2

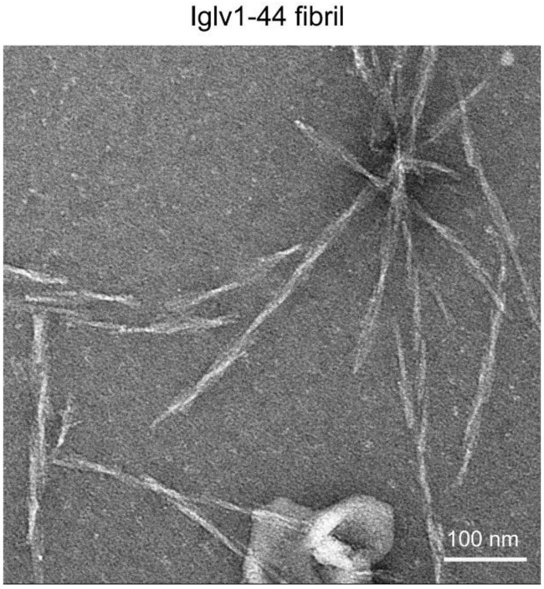

Supplementary Figure 2

Negative-staining TEM images reveal the characteristic helical symmetry of IGLV1-44 fibrils.

Supplementary Figure 3

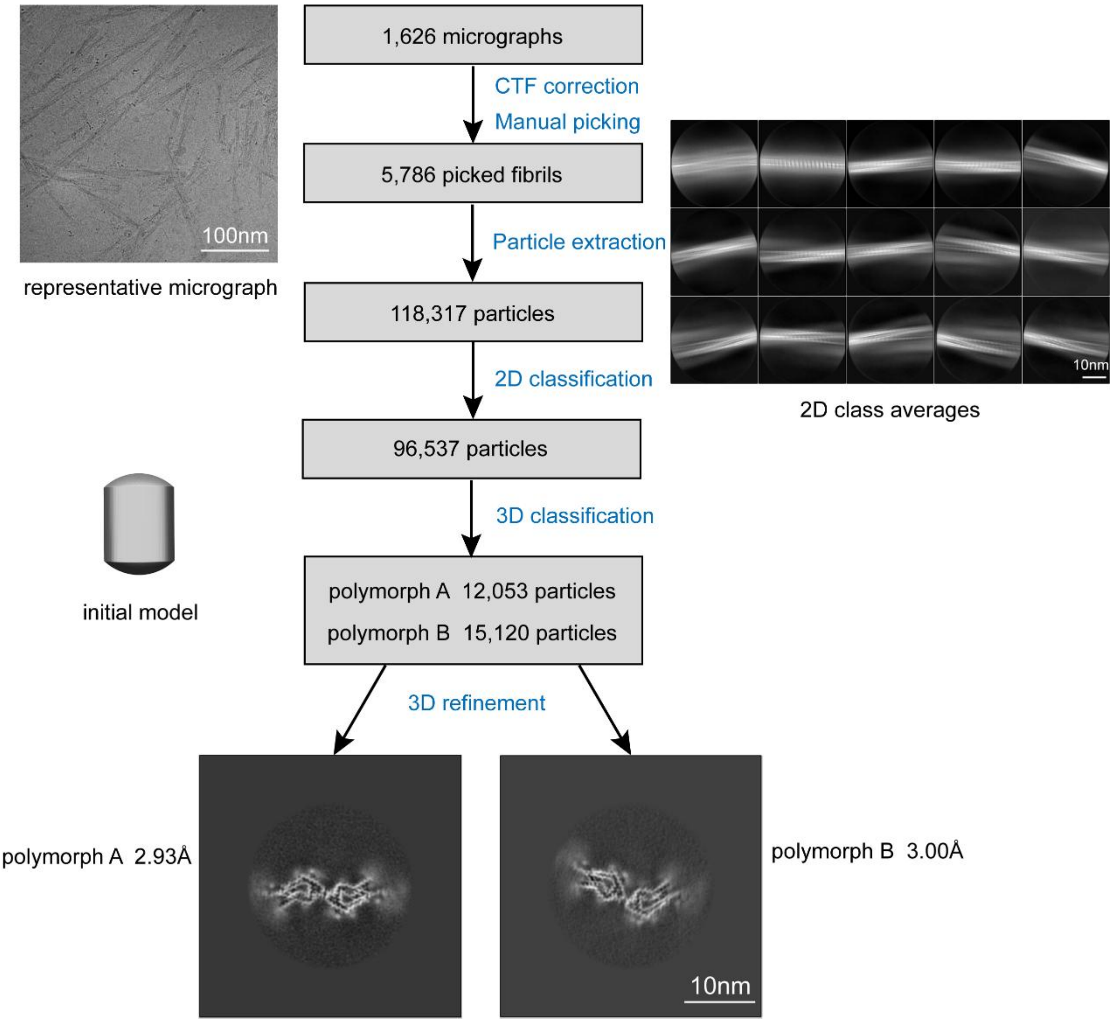

Supplementary Figure 3

Work flow of steps of the reconstruction process.

A representative micrograph and 2D class average are shown at the top left and top right, respectively. Scale bars are shown in the pictures. Data statistics, no. of micrographs, and no. of particles used in image processing are listed. Based on an initial reconstruction, 3D classification separated two subsets exhibiting different density paths. Through several further classification and refinement steps, the final reconstructions were obtained.

Supplementary Figure 4

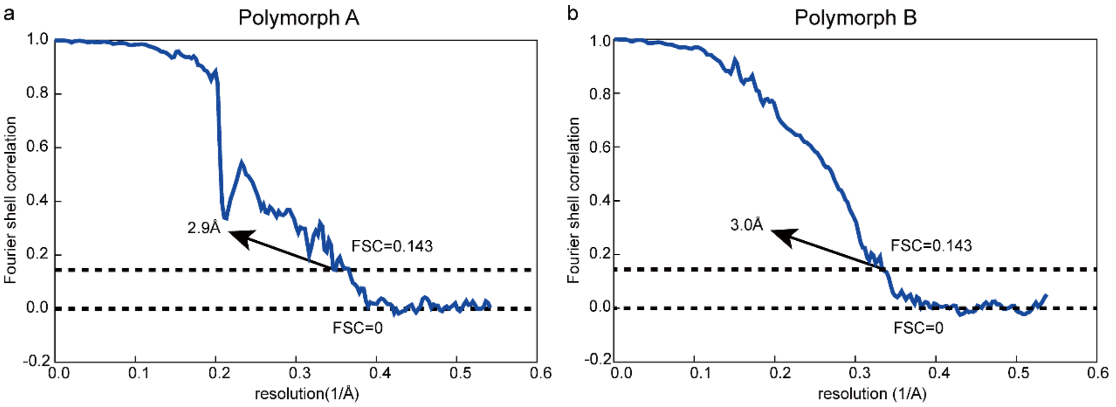

Supplementary Figure 4

FSC curve of the reconstructed density.

Supplementary Figure 5

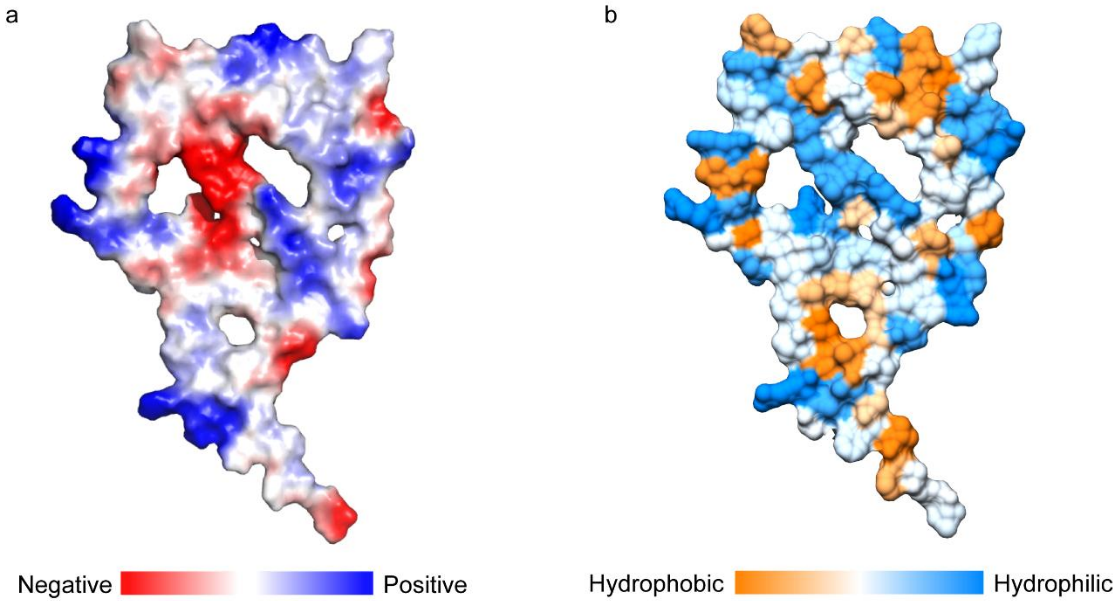

Supplementary Figure 5

Surface properties of the cardiac IGLV1-44 fibril (PDB:6IC3).

(a)Electrostatic surface representation of the fibril protein. Red indicates negative charge, blue positive, and white neutral. (b)Hydrophobic surface representation of the fibril protein. Orange indicates hydrophobic residues， blue hydrophilic, and white neutral.

Supplementary Figure 6

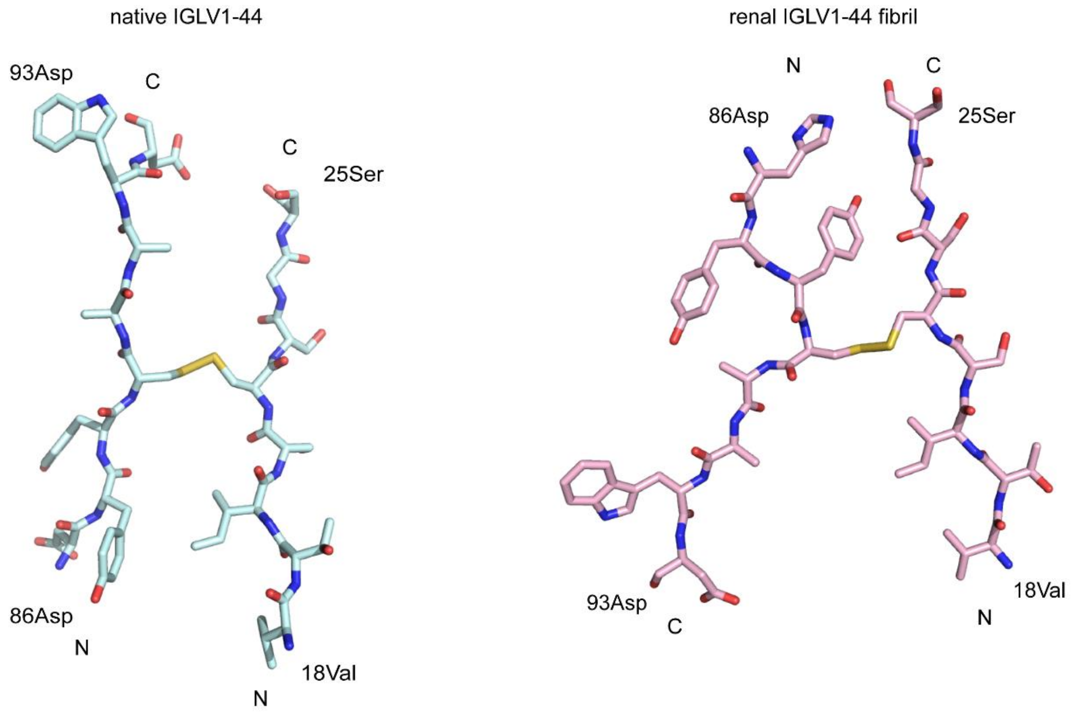

Supplementary Figure 6

Comparison of the disulfide bond of the native IGLV1-44 and renal IGLV1-44 fibril.

Part of the native structure (colored in pale cyan) and of the fibril state (colored in light pink) showing the conformational switch of segments 86–93 and 18–25 relative to one another around the protein disulfide bond.

Supplementary Table 1

Name	IGLV1-44 polymorph A	IGLV1-44 polymorph B
PDB ID	9X45	9V91
EMDB ID	EMD-66530	EMD-64858
**Data Collection**		
Magnification	130,000	130,000
Pixel size (Å)	0.93	0.93
Defocus Range (μm)	-0.7 to -1.5	-0.7 to -1.5
Voltage (kV)	300	300
Camera	Falcon 4i	Falcon 4i
Microscope	Titan Krios	Titan Krios
Exposure time	3.92	3.92
Dose rate	13.24	13.24
Total dose (e^-/Å)	52	52
**Reconstruction**		
Micrographs	1,626	1,626
Manually picked fibrils	5,786	5,786
Box size (pixel)	400	400
Inter-box distance (Å)	37.2	37.2
Segments extracted	118,317	118,317
Segments after Class2D	96,537	96,537
Segments after Class3D	12,053	15,120
Resolution (Å)	2.93	3.00
FSC threshold	0.143	0.143
Map sharpening B-factor (Å^2)	-46.07	-35.29
Helical rise (Å)	2.46	4.92
Helical twist (°)	-179.58	0.87
Symmetry imposed	C1	C1
**Atomic model**		
Non-hydrogen atoms	3,588	3,588
Protein residues	480	480
Ligands	0	0
r.m.s.d Bond lengths	0.004	0.004
r.m.s.d Bond angles	1.068	1.068
All-atom clashscore	19.21	18.63
Rotamer outliers	0.00%	0.00%
Ramachandran Outliers	0.00%	0.00%
Ramachandran Allowed	26.92%	26.92%
Ramachandran Favored	73.08%	73.08%

Supplementary Table 1

Statistics of cryo-EM data collection and refinement.
