# Supplementary material for: Cryo-EM structure of renal AL amyloid fibrils from a patient with λ1 light chain amyloidosis
Source: Nat Commun. 2025 Dec 17;17:848. doi: 10.1038/s41467-025-67556-0 (PMC12827339; doi:10.1038/s41467-025-67556-0)
Supplement: Supplementary file 2 — Reporting Summary [file 41467_2025_67556_MOESM2_ESM.pdf]

nature portfolio

Corresponding author(s): He Huang & Kun Zhao

Last updated by author(s): Nov 25, 2025

Reporting Summary

Nature Portfolio wishes to improve the reproducibility of the work that we publish. This form provides structure for consistency and transparency in reporting. For further information on Nature Portfolio policies, see our Editorial Policies and the Editorial Policy Checklist.

Statistics

For all statistical analyses, confirm that the following items are present in the figure legend, table legend, main text, or Methods section.

n/a	Confirmed	
☐	☒	The exact sample size (*n*) for each experimental group/condition, given as a discrete number and unit of measurement
☒	☐	A statement on whether measurements were taken from distinct samples or whether the same sample was measured repeatedly
☒	☐	The statistical test(s) used AND whether they are one- or two-sided<br>*Only common tests should be described solely by name; describe more complex techniques in the Methods section.*
☒	☐	A description of all covariates tested
☒	☐	A description of any assumptions or corrections, such as tests of normality and adjustment for multiple comparisons
☐	☒	A full description of the statistical parameters including central tendency (e.g. means) or other basic estimates (e.g. regression coefficient) AND variation (e.g. standard deviation) or associated estimates of uncertainty (e.g. confidence intervals)
☒	☐	For null hypothesis testing, the test statistic (e.g. *F*, *t*, *r*) with confidence intervals, effect sizes, degrees of freedom and *P* value noted<br>*Give P values as exact values whenever suitable.*
☒	☐	For Bayesian analysis, information on the choice of priors and Markov chain Monte Carlo settings
☒	☐	For hierarchical and complex designs, identification of the appropriate level for tests and full reporting of outcomes
☒	☐	Estimates of effect sizes (e.g. Cohen's *d*, Pearson's *r*), indicating how they were calculated

Our web collection on statistics for biologists contains articles on many of the points above.

Software and code

Policy information about availability of computer code

Data collection	EPU
Data analysis	Relion v3.0, MotionCor2 v1.2.1, Ctffind v4.1.8, COOT v0.8.9.2, PHENIX v1.20, UCSF Chimera v1.17.3, PEAKS Studio v13

For manuscripts utilizing custom algorithms or software that are central to the research but not yet described in published literature, software must be made available to editors and reviewers. We strongly encourage code deposition in a community repository (e.g. GitHub). See the Nature Portfolio guidelines for submitting code & software for further information.

Data

Policy information about availability of data

All manuscripts must include a data availability statement. This statement should provide the following information, where applicable:

- Accession codes, unique identifiers, or web links for publicly available datasets
- A description of any restrictions on data availability
- For clinical datasets or third party data, please ensure that the statement adheres to our policy

The reconstructed cryo-EM maps were deposited in the Electron Microscopy Data Bank with the accession codes EMD-66530 [https://www.ebi.ac.uk/pdbe/entry/emdb/EMD-66530] (polymorph A) and EMD-64858 [https://www.ebi.ac.uk/pdbe/entry/emdb/EMD-64858] (polymorph B). The coordinate files of the fibril models were deposited in the Protein Data Bank with the accession codes 9X45 [https://doi.org/10.2210/pdb9X45/pdb] (polymorph A) and 9V91 [https://doi.org/10.2210/pdb9v91/pdb] (polymorph B). The mass spectrometry raw data was deposited in ProteomeXchange database [http://proteomecentral.proteomexchange.org/cgi/GetDataset?ID=PXD066587] (accession number PXD066587). Source data are provided with this paper for the Supplementary Fig. 4.

Research involving human participants, their data, or biological material

Policy information about studies with human participants or human data. See also policy information about sex, gender (identity/presentation), and sexual orientation and race, ethnicity and racism.

Reporting on sex and gender	Sex or gender are not relevant for the purpose of this study
Reporting on race, ethnicity, or other socially relevant groupings	Race, ethnicity or socially relevant groupings are not relevant for the purpose of this study
Population characteristics	Age;sex;diagnosis<br>66 years old; male; major renal involvement
Recruitment	Selected based on clinical findings (Congo red staining to prove the presence of amyloid and immunostaining with antibodies specific for lambda light chains) at percutaneous kidney biopsy.
Ethics oversight	This study was approved by the Ethical Committee of the First Affiliated Hospital, Zhejiang University School of Medicine ([2025B]IIT Ethics Approval No.0278)

Note that full information on the approval of the study protocol must also be provided in the manuscript.

Field-specific reporting

Please select the one below that is the best fit for your research. If you are not sure, read the appropriate sections before making your selection.

☒ Life sciences ☐ Behavioural & social sciences ☐ Ecological, evolutionary & environmental sciences

For a reference copy of the document with all sections, see nature.com/documents/nr-reporting-summary-flat.pdf

Life sciences study design

All studies must disclose on these points even when the disclosure is negative.

Sample size	Sample size is one, as the fibrils were extracted from a single patient.
Data exclusions	Nodata were excluded.
Replication	Single patient. No replicate.
Randomization	Not relevant to the study. Amyloid deposits were analyzed from a single patient.
Blinding	Not relevant to the study. Amyloid deposits were analyzed from a single patient.

Reporting for specific materials, systems and methods

We require information from authors about some types of materials, experimental systems and methods used in many studies. Here, indicate whether each material, system or method listed is relevant to your study. If you are not sure if a list item applies to your research, read the appropriate section before selecting a response.

Materials & experimental systems

n/a	Involved in the study
☒	☐ Antibodies
☒	☐ Eukaryotic cell lines
☒	☐ Palaeontology and archaeology
☒	☐ Animals and other organisms
☒	☐ Clinical data
☒	☐ Dual use research of concern
☒	☐ Plants

Methods

n/a	Involved in the study
☒	☐ ChIP-seq
☒	☐ Flow cytometry
☒	☐ MRI-based neuroimaging

Plants

Seed stocks	*Report on the source of all seed stocks or other plant material used. If applicable, state the seed stock centre and catalogue number. If plant specimens were collected from the field, describe the collection location, date and sampling procedures.*
Novel plant genotypes	*Describe the methods by which all novel plant genotypes were produced. This includes those generated by transgenic approaches, gene editing, chemical/radiation-based mutagenesis and hybridization. For transgenic lines, describe the transformation method, the number of independent lines analyzed and the generation upon which experiments were performed. For gene-edited lines, describe the editor used, the endogenous sequence targeted for editing, the targeting guide RNA sequence (if applicable) and how the editor was applied.*
Authentication	*Describe any authentication procedures for each seed stock used or novel genotype generated. Describe any experiments used to assess the effect of a mutation and, where applicable, how potential secondary effects (e.g. second site T-DNA insertions, mosiacism, off-target gene editing) were examined.*
